# Der mögliche Nutzen künstlicher Intelligenz in einem organisierten bevölkerungsbezogenen Screeningprogramm: Erste Ergebnisse und Ausblick

**DOI:** 10.1007/s00117-024-01345-6

**Published:** 2024-07-17

**Authors:** R. Morant, A. Gräwingholt, J. Subelack, D. Kuklinski, J. Vogel, M. Blum, A. Eichenberger, A. Geissler

**Affiliations:** 1 Krebsliga Ostschweiz, Flurhofstrasse 7, 9000 St. Gallen, Schweiz; 2 Radiologie am Theater, 33098 Paderborn, Deutschland; 3 School of Medicine, Lehrstuhl für Gesundheitsökonomie, -Politik und -Management, Universität St. Gallen, 9000 St. Gallen, Schweiz

**Keywords:** Mammographiescreening, Brustkrebs, Maschinelles Lernen, Intervallkarzinome, Schweiz, Mammography screening, Breast cancer, Machine learning, Interval cancer, Switzerland

## Abstract

**Hintergrund:**

Dank Mammographie-Screening-Programmen (MSP) kann Brustkrebs erwiesenermaßen in früheren Stadien entdeckt werden, was weniger eingreifende Therapien erlaubt und zu einem besseren Überleben führt. Kritisch beurteilt werden die beträchtliche Zahl der Intervallkarzinome (IBC) und zusätzlich notwendige Abklärungen, bei denen sich in der Mehrzahl erweist, dass kein Karzinom vorliegt.

**Fragestellung:**

In den letzten Jahren wurden von Firmen und Universitäten mittels maschinellem Lernen (ML) leistungsfähige Algorithmen entwickelt, welche erstaunliche Fähigkeiten zum Lesen von Mammographien zeigen. Können dadurch MSP qualitativ verbessert werden?

**Methode:**

Mittels der Software ProFound AI® (iCAD, Nashua, NH, USA) wurden retrospektiv die ursprünglichen Screening-Mammographien von 251 Fällen mit Intervallkarzinom untersucht und die Resultate (Case-Score, Risk-Score) mit denen einer Kontrollgruppe verglichen. Darüber hinaus wurde die relevante aktuelle Literatur studiert.

**Ergebnisse:**

Die Verteilung des Case-Score wie auch des Risk-Score der Mammographien mit späterem IBC war signifikant zu höherem Risiko verschoben im Vergleich zur Kontrolle, ähnlich wie in anderen Studien.

**Schlussfolgerung:**

Retrospektive Studien, wie auch eigene Daten zeigen, dass möglicherweise künstliche Intelligenz (KI) in Zukunft das Vorgehen bei MSP ändern wird in Richtung personalisiertem Screening, mit deutlicher Entlastung der Radiologen, weniger Abklärungen und einer verminderten Anzahl von IBC. Für eine solche Umsetzung braucht es die Resultate prospektiver Studien.

## Abstract (en)

**Background:**

Mammography screening programs (MSP) have shown that breast cancer can be detected at an earlier stage enabling less invasive treatment and leading to a better survival rate. The considerable numbers of interval breast cancer (IBC) and the additional examinations required, the majority of which turn out not to be cancer, are critically assessed.

**Objective:**

In recent years companies and universities have used machine learning (ML) to develop powerful algorithms that demonstrate astonishing abilities to read mammograms. Can such algorithms be used to improve the quality of MSP?

**Method:**

The original screening mammographies of 251 cases with IBC were retrospectively analyzed using the software ProFound AI® (iCAD) and the results were compared (case score, risk score) with a control group. The relevant current literature was also studied.

**Results:**

The distributions of the case scores and the risk scores were markedly shifted to higher risks compared to the control group, comparable to the results of other studies.

**Conclusion:**

Retrospective studies as well as our own data show that artificial intelligence (AI) could change our approach to MSP in the future in the direction of personalized screening and could enable a significant reduction in the workload of radiologists, fewer additional examinations and a reduced number of IBCs; however, the results of prospective studies are needed before implementation.

In den letzten 30 Jahren haben sich Mammographie-Screening-Programme (MSP) weltweit zunehmend verbreitet. Abklärungen wurden in zertifizierten Brustzentren besser organisiert, und die chirurgischen, radiotherapeutischen und medizinischen Behandlungsmöglichkeiten wurden kontinuierlich verbessert. Das Überleben von Brustkrebspatientinnen hat sich in der Folge trotz der steigenden Inzidenz von Brustkrebs deutlich gebessert, international [1] aber auch in Deutschland [2] und in der Schweiz [3].

Der Einsatz künstlicher Intelligenz (KI) hält auch in der Medizin immer mehr Einzug, speziell in der Radiologie und Mammographie-Diagnostik. Daher wird in der vorliegenden Arbeit dargelegt, wie sich der Einsatz von KI auf die Brustkrebsfrüherkennungsprogramme in Zukunft auswirken könnte.

## Künstliche Intelligenz in der Mammographie-Diagnostik

Erste Versuche [4] mit CAD-Systemen („computer-aided diagnostic“, computerunterstützte Diagnostik) in der Mammographie-Diagnostik wurden schon kurz vor der Jahrtausendwende durchgeführt [5]. Die verwendeten Algorithmen beruhten auf Expertenwissen von tumorverdächtigen Bezirken, wie unregelmäßig begrenzten Verdichtungen, Spikulae und Mikrokalk.

Im praktischen Einsatz zeigte sich allerdings, dass der Nutzen solcher Programme recht eingeschränkt war, die ursprünglichen Erwartungen nicht erfüllt werden konnten und häufig falsch-positive Befunde angezeigt wurden. Diese CAD-Systeme konnten die Radiologen lediglich unterstützend auf möglicherweise ungenügend beachtete Bezirke hinweisen, da das Programmieren auf Grund von Expertenwissen technisch an die Grenzen gestoßen war.

Dies änderte sich durch Technologiesprünge in den folgenden Jahren. Ab 2012 wurde „Deep Learning“ zur Entwicklung der diagnostischen Software eingesetzt. Die Computer wurden mit riesigen Datensätzen von unauffälligen Mammographien und von solchen mit Karzinomen trainiert. Die Software für dieses maschinelle Lernen (ML) ist in Analogie zu neuronalen Netzwerken auf unterschiedlich vielen Lagen von verborgenen Schichten aufgebaut. Die ML-basierten Algorithmen konnten die Diagnostik erheblich verbessern. Zu dieser Entwicklung haben immer leistungsfähigere Computer wesentlich beigetragen.

Ein Nachteil solcher Computerprogramme ist, dass den Menschen bei dieser Art von Maschinenlernen verborgen bleibt, warum der Algorithmus auffällige Bezirke erkennt. Somit bleibt eine sorgfältige Überprüfung der KI-Ergebnisse in Studien unverzichtbar, und es muss bekannt sein, welche Trainingsdaten verwendet wurden. Diese können sich je nach Alter der Probandinnen, Weltgegend, genetischem Hintergrund aber auch der Qualität unterscheiden, was die Übertragbarkeit auf andere Personengruppen einschränken kann.

Es gibt zurzeit eine Vielzahl von Algorithmen, die an Universitäten, aber auch durch kommerziell tätige Firmen entwickelt wurden, und die in der Forschung und Praxis verwendet werden.

Das Ergebnis solcher KI-Analysen sind quantitative Scores, welche die Wahrscheinlichkeit angeben, dass ein vom Computer als auffällig erkannter Bezirk ein Karzinom ist. Je nach Entwickler werden die Resultate in unterschiedlicher Weise angegeben, als Zahl zwischen 0 und 100, oder 0 bis 1. Zudem werden die auffälligen Stellen in der Mammographie grafisch markiert.

Unterschieden werden Scores, welche sich auf eine einzelne verdächtige Läsion beziehen („lesion score“) von solchen, welche sich auf die ganze Brust beziehen („case score“) und das Ergebnis von mehreren verdächtigen Bezirken sein können.

Diese Programme erweisen sich in der Praxis als erstaunlich beeindruckend und werden oft bereits in Radiologie-Instituten unterstützend als Zweitbefunder eingesetzt.

KI kann aus der Struktur und Dichte des Brustgewebes sowie dem Vorhandensein von Mikrokalk und Asymmetrien darüber hinaus das Risiko einer zukünftigen Karzinomdiagnose vorhersagen. Dieser Risk-Score inkludiert in seine Berechnungen weitaus mehr Faktoren als die Brustdichte. Diese ist ein anerkannter Risikofaktor für Brustkrebs (so verdoppelt sich das Risiko bei ACR D im Vergleich zu ACR B; [6]) und ist verantwortlich für eine verminderte Sensitivität der Mammographie [7]. KI-Algorithmen können die Brustdichte konsistenter bestimmen, als dies durch die Einschätzung einzelner Radiologen möglich ist. Ein quantitativer Risk-Score einer Mammographie wird von der Firma iCAD kommerziell angeboten. Das Resultat ist eine Risikoklassifizierung, welche die Wahrscheinlichkeit einer Karzinomdiagnose innerhalb der nächsten 2 Jahre in niedrig, durchschnittlich, leicht oder stark erhöht einteilt.

In einer Studie wurde das Brustkrebsrisiko von als normal befundeten Mammographien von fünf unterschiedlichen akademischen und kommerziellen Algorithmen beurteilt. Das Risiko, dass Brustkrebs innerhalb von 5 Jahren diagnostiziert wird, konnte besser und weniger aufwendig vorhergesagt werden als mit den bisherigen Risikomodellen, welche auf klinischen Faktoren basieren [8]. In einem englischen MSP des National Health Service (NHS) wurden Mammographien untersucht, welche 3 Jahre vor einer diagnostischen Screening-Mammographie erstellt worden waren [9]. Verglichen wurden 4 Open-Source-Algorithmen, wovon einer als Risikoalgorithmus entwickelt wurde, die anderen zur Krebsdiagnose. Auch diese letzteren zeigten zusätzlich eine prognostische Aussagekraft. Möglicherweise wurden damit minimale Zeichen eines vom menschlichen Auge noch nicht wahrnehmbaren Karzinoms entdeckt.

In verschiedenen Studien zeigte sich, dass die Sensitivität von KI bei der Entdeckung eines Karzinoms mit der Sensitivität von vielen Radiologen vergleichbar ist, aber schlechter sein kann als die der besten Radiologen [10]. Wenn einem Radiologen die Resultate einer Software zur Verfügung stehen, wird seine Performance erhöht.

Ein Nachteil aller bis jetzt erhältlichen Programme ist, dass Voraufnahmen noch nicht in die Beurteilung durch die KI miteinbezogen werden. Bezüglich des Einsatzes von KI-Algorithmen bei der Tomosynthese existieren bislang zu wenige Daten für eine abschließende Beurteilung. Die optimale Implementation dieser Systeme in die operativen Prozesse von MSP ist bisher noch wenig erforscht.

## Mammographie-Screening-Programme in Europa

Seit den 1980er-Jahren wurden in vielen europäischen Ländern MSP eingeführt, zuerst in Finnland und Schweden. In der Schweiz wurden in der Westschweiz um das Jahr 2000 mehrere MSP gestartet, ab 2010 mit St. Gallen auch in der Deutschschweiz [11]. Es gibt weiterhin einige Kantone ohne MSP.

Mortalitätsvergleiche von Teilnehmerinnen, deren Karzinome innerhalb oder außerhalb eines Screeningprogramms entdeckt wurden, zeigten deutliche Überlebensvorteile für die MSP-entdeckten Karzinome, zwischen 25 und 40 %, international und auch in der Schweiz [3]. Diese Effekte sind größer als in den alten randomisierten Studien. Es konnte sogar eine Abnahme der Brustkrebsmortalität in der gesamten gescreenten Altersgruppe gezeigt werden [14, 15].

MSP haben einen positiven Einfluss auf die Lebensqualität durch eine geringere Anzahl von Mastektomien [12] und weniger häufigem Einsatz von Chemotherapie.

Der gesundheitspolitische und ökonomische Nutzen [13] von MSP ist in Europa weitgehend anerkannt, wenn auch in Teilaspekten immer wieder umstritten oder kritisiert (Intervallkarzinome, zu viele weitere Abklärungen).

Es ist daher von großem Interesse, festzustellen, ob und wie die Effektivität von MSP verbessert werden kann.

## Erste Resultate des Einsatzes von KI in MSP

Erste Ergebnisse des Einsatzes von KI-Algorithmen in MSP sind auf Basis von retrospektiven Untersuchungen für verschiedene Softwareprodukte in unterschiedlichen Settings zu finden. Zudem werden vermehrt prospektive Studien aufgesetzt, von denen einzelne Zwischenergebnisse berichtet wurden. Im Folgenden wird auf die Ergebnisse von verschiedenen europäischen Studien eingegangen.

Eine retrospektive holländische Studie hat 2023 eine Screeningpopulation von 272.008 Mammographien mit der KI Transpara® (Nijmegen, Niederlande) untersucht. Es zeigte sich, dass bei einem Einsatz von KI als alleinigem Befunder sowohl die Sensitivität als auch die Spezifizität gegenüber dem radiologischen Erstbefunder verringert würde. Bei einem kombinierten Einsatz von KI und einem menschlichen Befunder würde sich hingegen im Vergleich zum üblichen Vorgehen mit zwei Befundern eine leicht höhere Sensitivität und größere Entdeckungswahrscheinlichkeit von späteren Intervallkarzinomen (IBC) ergeben, bei einer allerdings etwas niedrigeren Spezifizität.

Eine norwegische Studie [16] untersuchte gleichfalls mit Transpara® retrospektiv Mammographien von 949 beim Screening diagnostizierte Mammakarzinomen (SBC) und zusätzlich von 305 Fällen, bei denen später ein IBC entdeckt wurde. Bei SBC wurde der höchste Score von 10 (Skala von 0–10) in 92,7 % der Mammographien gefunden, und in 40 % der Fälle mit späterem IBC, was eine höhere Sensitivität als die eines einzelnen Radiologen bedeutet. In 41,9 % dieser SBC wurde schon in der vorangegangenen Screening-Mammographie der höchste Score von 10 gefunden. Das weist darauf hin, dass KI die Diagnose von Tumoren in noch früherem Stadium erlauben könnte. Die hohe Sensitivität des Computerprogramms zeigte sich auch bei sehr hoher Brustdichte, im Gegensatz zur Beurteilung durch Radiologen. So hatten selbst bei den Fällen mit der höchsten Brustdichte alle SBC und 48,6 % der IBC den höchsten KI-Score von 10. Dies würde bedeuten, dass durch den Einsatz der KI die Sensitivität im Programm deutlich verbessert werden könnte (von 62,8 % auf 80,9 %). In einem späteren Kongressbericht am ECR 2024 berichteten die Autoren allerdings, dass in einer retrospektiven Konsensuskonferenz der Screening-Mammographien mit sehr dichter Brust und späterem Intervallkarzinom trotz einem KI-Score von 10 häufig keine zielführende weitere Abklärung möglich war, d. h. dass der Wert der KI zur Verhinderung von IBC weiterhin nicht gesichert erscheint.

Der Risk-Score von ProFound AI® wurde retrospektiv in 3 Screening-Populationen in Spanien, Deutschland und Italien untersucht [17]. In diesen Studien wurden 7812 normale Mammographien mit solchen von 739 Frauen, bei denen später Brustkrebs aufgetreten war, verglichen. Es zeigte sich, dass das relative Risiko einer Brustkrebsdiagnose der Frauen mit Mammographien mit einem erhöhten Risk-Score 6,7-mal höher war. Solche Studien können Hinweise für die Gestaltung eines risikoadaptierten MSP geben.

In der prospektiven schwedischen KARMA-Studie wird zusätzlich zu den radiologischen Risikofaktoren für die Entstehung eines Brustkrebses ein sog. Polygenic Risk Score (PRS) hinzugezogen. Dieser berechnet mittels einer genetischen Untersuchung das Risiko einer zukünftigen Brustkrebsdiagnose. Dieser PRS setzt sich aus der Summe von Teilrisiken einer Vielzahl von Genvarianten zusammen, welche einzeln jeweils nur eine leichte Erhöhung oder Erniedrigung von Brustkrebserkrankungen mit sich bringen [18]. PRS werden auch in der Schweiz in einer multizentrischen Studie von Leo et al. untersucht. In einer schwedischen Studie zeigte sich, dass sogar erstgradig Verwandte von Brustkrebspatientinnen mit einem Hochrisiko-PRS ein höheres Brustkrebsrisiko haben [19].

Retrospektive Studien haben naturgemäß einige Limitationen, sodass die Resultate prospektiver Studien entscheidend sein werden [20]. So vergleicht die Gemini-Studie randomisiert bei 200.000 Teilnehmerinnen ein konventionelles Vorgehen mit Doppelbefundung mit einer Interventionsgruppe, in der eine KI (Kheiron und Alphabet Inc) als Zweitleser eingesetzt wird.

Eine weitere prospektive Studie zeigte, dass das Ersetzen eines Radiologen durch KI (Insight MMG®, Korea) die Entdeckung von Karzinomen in einem schwedischen MSP im Vergleich zu zwei menschlichen Befundern in einer Nichtunterlegenheitsstatistik nicht verschlechterte [21], und zudem die Zahl weiterer Abklärungen (Recall) verringerte trotz 21 % mehr Fällen in der Konsensuskonferenz. Selbst wenn KI als alleiniger Befunder eingesetzt würde, wären nicht weniger Karzinome entdeckt worden, aber 47 % weniger Recall erfolgt.

Eine andere schwedische Studie randomisierte zwischen einer Gruppe, bei der KI bei einem Score bis 9 (Transpara®) mit nur einem Befunder eingesetzt wurde und nur beim höchsten Score 10 mit zusätzlich 2 Radiologen. Die Krebsentdeckungsrate war in der Interventionsgruppe etwas höher (keine statistische Signifikanz), die Arbeitsbelastung der Radiologen sank um 44,3 % [22].

## Eigene Erfahrungen der Krebsliga Ostschweiz mit dem Einsatz von KI bei der Beurteilung von Intervallkarzinomen

Die Krebsliga Ostschweiz betreibt im Auftrag von 6 Kantonen das Brustkrebsfrüherkennungsprogramm „donna“, welches zuerst im Kanton St. Gallen im Jahr 2010 gestartet wurde. In Zusammenarbeit mit der School of Medicine der Universität St. Gallen wurden die bis anhin beobachteten Intervallkarzinome in St. Gallen und Graubünden von 2010 bis 2019 identifiziert, biologisch beschrieben und retrospektiv mit der Software ProFound AI® von iCAD untersucht. Die präliminären Resultate wurden am Jahrestreffen der Schweizerischen Senologiegesellschaft im September 2023 in Zürich sowie am ECR in Wien im März 2024 vorgestellt. Die finalen Resultate der Studie werden dieses Jahr noch publiziert. Ziel dieser Studie ist eine Verringerung der Anzahl an IBC, welche im Vergleich zu SBC in einem prognostisch ungünstigeren höheren Stadium auftreten, maligner sind (höheres KI67, häufiger HER2+++), und zu einer niedrigeren Überlebensrate führen.

**Abb. 1:**
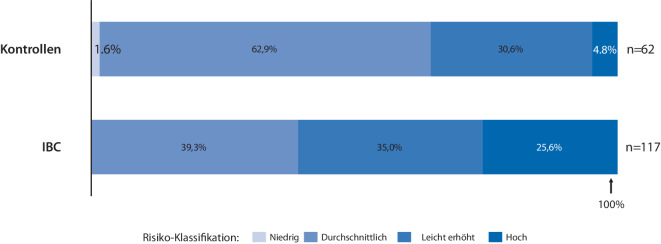
Unauffällig befundete Screening-Mammographien von Frauen, die später mit IBC diagnostiziert wurden, haben eine Verteilung des Risk-Scores zu deutlich höherem Risiko hin. *IBC* Intervallkarzinom

251 IBC und eine kleine Vergleichsgruppe von SBC der letzten 10 Jahre wurden durch die KI-Software erneut befundet. Sowohl der Case-Score als auch der Risk-Score (Abb. 1) der zuvor von Radiologen als unauffällig beurteilten Screening-Mammographien von Teilnehmerinnen mit späteren IBC waren signifikant höher im Vergleich zu Mammographien von Frauen, bei denen in den folgenden 2 Jahren kein IBC diagnostiziert wurde (22,7 % IBC haben einen Case-Score von > 60, im Vergleich zu 0 % in einem kleinen Vergleichskollektiv). Je nach der gewählten Höhe des Schwellenwerts des Case-Scores verändern sich Sensitivität und Spezifität des Resultats in umgekehrter Richtung. Selbst bei einem hohen Grenzwert des Case-Scores von 60 oder einem als „hoch“ eingestuften Risk-Score kämen 14 % der IBC, welche bisher nicht für eine Konsensuskonferenz qualifiziert waren, nun in eine solche Konferenz, d. h. sie würden möglicherweise früher abgeklärt.

Dieses Resultat deutet darauf hin, dass durch Einsatz von KI die Anzahl der IBC verringert werden könnte. Den optimalen Grenzwert der Sensitivität der Scores ohne eine zu hohe Belastung durch zusätzliche Konsensuskonferenzen werden wir in einer weiteren retrospektiven Untersuchung analysieren.

Eine frühere Studie mit 29 auswertbaren Intervallkarzinomen eines deutschen MSP ergab, dass die KI (ProFound AI® von iCAD) in 48 % die jeweils frühere Screening-Mammographie als auffällig bezeichnete, was auch auf das Potenzial zur Verringerung von IBC hinwies [23].

## Ausblick und Ziel

Ziele des Einsatzes von KI in MSP sind eine Verbesserung der Qualität bei gleichzeitiger Verringerung der Arbeitsbelastung der Radiologen. Dies bedeutet eine erhöhte Sensitivität, eine verminderte Abklärungsrate und weniger Intervallkarzinome.

KI könnte dem weltweiten Mangel an qualifizierten Radiologen entgegenwirken und helfen, kostengünstig MSP auch in weiteren Weltgegenden durchzuführen. KI kann aktuell in einem MSP zwar (noch) nicht beide Radiologen gleichwertig ersetzen [24], es kann jedoch ein risikoadaptiertes Vorgehen diskutiert werden. Beispielsweise könnte bei Mammographien mit gemäß KI sehr geringem Krebsrisiko eine zusätzliche Lesung durch einen Radiologen entfallen, und in einer höheren Risikostufe eine Beurteilung nur noch durch einen statt zwei Radiologen erfolgen. Allerdings hat die EU (ECIBC: European Commission Initiative on Breast Cancer) im Juli 2023 empfohlen, in MSP die übliche Doppelbefundung noch nicht durch die KI und nur noch einen menschlichen Befund zu ersetzen. Die konkrete Ausgestaltung zukünftiger Programme ist also noch offen und hängt vom Ergebnis prospektiver Studien in Screeningsettings ab.

Eine weitere Stoßrichtung ist die Einführung risikoadaptierter Screeninguntersuchungen. So könnten Programme aufgesetzt werden, in denen Frauen mit einem erhöhtem Risk-Score gemäß KI zusätzlich oder alternativ eine andere Diagnostik, wie Ultraschall, Tomosynthese oder Magnetresonanz(MR)-Untersuchung erhalten und/oder das Screeningintervall reduziert wird. Eine MR-Mammographie wird bei sehr hoher Brustdichte bereits durch aktuelle Empfehlungen unterstützt (EUSOBI, 2022; [25]). Andererseits könnte bei Frauen mit sehr geringem Risiko das Screeningintervall von heute meist 2 Jahren z. B. auf 3 Jahre erhöht werden. Bei der Planung eines risikoadaptierten Screenings können neben radiologischen Faktoren auch Risiken wie Genetik, Familienanamnese oder Hormonbehandlungen einbezogen werden.

Zu vielen anderen Fragen gehört auch die Unsicherheit, wie das Wissen um die Ergebnisse der KI die Radiologen und die Konsensuskonferenz beeinflussen wird [26]. Auch die Effektivität der unterschiedlichen Algorithmen muss vergleichend untersucht werden.

### Kernaussagen.

- Vorliegende Untersuchungen legen nahe, dass künstliche Intelligenz (KI) die Sensitivität eines MSP erhöhen und die Zahl der Intervallkarzinome verringern könnte.
- Durch Triage und Ersatz des Zweitlesers könnte in Mammographie-Screening-Programmen (MSP) eine Reduktion des Workloads von Radiologen um 30−40 % bei gleichbleibender Sensitivität erreicht werden.
- Der Beweis für die Effektivität von KI in MSP muss in prospektiven Studien erfolgen.

## Fazit für die Praxis

Es besteht die berechtigte Hoffnung, dass durch Einsatz von KI MSP effektiver und durch Einsparung von Befunderzeit auch effizienter und günstiger gestaltet werden können.

## Abkürzungen

CAD: „Computer-aided diagnostic“
IBC: Intervallkarzinom
KI: Künstliche Intelligenz
ML: Maschinelles Lernen
MSP: Mammographie-Screening-Programm
PR: Polygenic Risk Score
